# Overexpression of *PvPin1*, a Bamboo Homolog of *PIN1-Type Parvulin 1*, Delays Flowering Time in Transgenic *Arabidopsis* and Rice

**DOI:** 10.3389/fpls.2017.01526

**Published:** 2017-09-08

**Authors:** Zhigang Zheng, Xiaoming Yang, Yaping Fu, Longfei Zhu, Hantian Wei, Xinchun Lin

**Affiliations:** ^1^State Key Laboratory of Subtropical Silviculture, Zhejiang Agriculture and Forestry University Hangzhou, China; ^2^State Key Laboratory of Rice Biology, China National Rice Research Institute Hangzhou, China

**Keywords:** *Phyllostachys violascens*, peptidylprolyl *cis/trans* isomerases, flowering, repressor, ecotopic expression, abscisic acid, methyl jasmonate

## Abstract

Because of the long and unpredictable flowering period in bamboo, the molecular mechanism of bamboo flowering is unclear. Recent study showed that *Arabidopsis* PIN1-type parvulin 1 (Pin1At) is an important floral activator and regulates floral transition by facilitating the *cis/trans* isomerization of the phosphorylated Ser/Thr residues preceding proline motifs in suppressor of overexpression of CO 1 (SOC1) and agamous-like 24 (AGL24). Whether bamboo has a *Pin1* homolog and whether it works in bamboo flowering are still unknown. In this study, we cloned *PvPin1*, a homolog of *Pin1At*, from *Phyllostachys violascens* (Bambusoideae). Bioinformatics analysis showed that PvPin1 is closely related to Pin1-like proteins in monocots. *PvPin1* was widely expressed in all tested bamboo tissues, with the highest expression in young leaf and lowest in floral bud. Moreover, *PvPin1* expression was high in leaves before bamboo flowering then declined during flower development. Overexpression of *PvPin1* significantly delayed flowering time by downregulating *SOC1* and *AGL24* expression in *Arabidopsis* under greenhouse conditions and conferred a significantly late flowering phenotype by upregulating *OsMADS56* in rice under field conditions. PvPin1 showed subcellular localization in both the nucleus and cytolemma. The 1500-bp sequence of the *PvPin1* promoter was cloned, and *cis*-acting element prediction showed that ABRE and TGACG-motif elements, which responded to abscisic acid (ABA) and methyl jasmonate (MeJA), respectively, were characteristic of *P. violascens* in comparison with *Arabidopsis*. On promoter activity analysis, exogenous ABA and MeJA could significantly inhibit *PvPin1* expression. These findings suggested that *PvPin1* may be a repressor in flowering, and its delay of flowering time could be regulated by ABA and MeJA in bamboo.

## Introduction

The transition from vegetative to reproductive growth must start at an appropriate time in flowering plants for producing progeny and perpetuating the species. Proper timing of flowering (or “heading date" in cereals) is controlled by environmental signals (Putterill et al., 2004; Brambilla and Fornara, 2013) and internal signals (Jack, 2004; Jarillo and Piñeiro, 2011). *Arabidopsis thaliana* as the model plant for eudicots has four main pathways involved in flowering control: photoperiod, vernalization, autonomous, and gibberellic acid (Simpson and Dean, 2002). Rice, a short-day and the model plant species for monocots, has photoperiod and *rice indeterminate 1* (*RID1*) pathways (Izawa, 2007; Wu et al., 2008).

Bamboo is a kind of widespread, fast-growing, renewable, and environmental-enhancing resource, whose industry contributes to providing food, building materials, and increasing the income for 2.2 billion people in the world (Chen, 2003). Bamboo products such as bamboo shoots, furniture, flooring, charcoal, beverages, and cosmetic are being used and traded by half of the world’s population (Chen, 2003). Although the bamboo industry is increasing in importance for poverty alleviation and economic development (Chen, 2003), bamboo flowering will make nothing left to these advantages because bamboo usually dies after flowering. In addition, it is difficult to analyze the phenomenon of bamboo flowering because of its unpredictability and long juvenile phase (Franklin, 2004). To surmount these problems, the genome of *Phyllostachys edulis* (synonym *Phyllostachys heterocycla*) and the transcriptomes of *P. edulis*, *Bambusa oldhamii*, *B. edulis*, and *Dendrocalamus latiflorus* have been sequenced, and numerous genes related to bamboo flowering were reported (Lin et al., 2010; Zhang et al., 2012; Peng et al., 2013; Gao et al., 2014; Shih et al., 2014; Zhao et al., 2014). As well, *P. edulis* and *D. latiflorus* contain novel miRNAs playing important roles in regulating bamboo flowering (Gao et al., 2015; Zhao et al., 2015). In addition, Louis et al. (2015) used proteomics to find that elements of stress, mobile genetics, and signal transduction cross-talk were associated with sporadic flowering of bamboo. Undoubtedly, these results provide the basis for understanding the roles of genes involved in bamboo flowering but need further experimental evidence.

The function of flowering genes has been heavily investigated in both *Arabidopsis* and rice; the results can provide some enlightenment on bamboo flowering. Recent study of *Arabidopsis* PIN1-type parvulin 1 (Pin1At) showed phosphorylation-dependent prolyl *cis/trans* isomerization of key transcription factors as an important flowering regulatory mechanism (Wang et al., 2010). In the 1980s, peptidylprolyl *cis/trans* isomerases (PPIases) were discovered (Fischer et al., 1983). Peptidylprolyl *cis/trans* isomerases act as enzymes catalyzing incongruous *cis/trans* isomerization of the peptide bonds preceding a proline residue to assist the client protein folding and restructuring (Kiefhaber et al., 1990; Hunter, 1998). There are four subfamilies of PPIases: FK506 binding proteins, cyclophilins, parvulins, and PP2A phosphatase activator (Lu and Zhou, 2007). Pin1, a member of the parvulin family of PPIases, is unique among the parvulin family because it functions by specifically recognizing phosphorylated Ser/Thr residues preceding proline (pSer/Thr-Pro) and catalyzing the conformational change of the phosphorylated substrates (Ranganathan et al., 1997; Hsu et al., 2001; Pastorino et al., 2006). Protein structure analysis showed that Pin1 in humans comprises an N-terminal WW regulatory domain and a C-terminal PPIase domain (Schiene-Fischer, 2015), and both domains can bind specifically to phospho-Ser/Thr-Pro-containing sequences (Yaffe et al., 1997; Lu et al., 1999). Pin1’s regulation of phosphorylation-dependent prolyl *cis/trans* isomerization has been found essential for cell growth and division, DNA repair, apoptosis, and transcription (Hanes et al., 1989; Hani et al., 1995; Lu et al., 1996).

*Pin1At* was the first identified PIN1-type PPIase from *Arabidopsis* (Landrieu et al., 2000; He et al., 2004); since then, several *Pin1* plant homologs from *Glycine max*, *Lycopersicon esculentum*, and *Malus domestica* have been cloned (Landrieu et al., 2000; Metzner et al., 2001; Yao et al., 2001; Wang et al., 2010). Unlike Pin1 in human and its homolog in yeast (Hanes et al., 1989; Hani et al., 1995; Lu et al., 1996), PIN1-type PPIases in plants have only one PPIase domain with four additional amino acids but without a WW domain (Yao et al., 2001), and except *Pin1At*, their function is still unknown.

Bamboo has unique characteristics in flowering. To determine whether bamboo has a *Pin1* homolog and whether it works in bamboo flowering, we isolated a *Pin1* homolog from *Phyllostachys violascens* (Lei bamboo) and named it *PvPin1*. Lei bamboo is widely distributed in southern China and has high economic value because of its delicious shoots. The income for intensively managed Lei bamboo forest is about 20 times that for rice (Song et al., 2011). However, shoot production of Lei bamboo forest decreases sharply during flowering. *PvPin1* from Lei bamboo was studied by analyzing its sequence structure, expression pattern, and phenotypes of transgenic *Arabidopsis* and rice. Unlike *Pin1At*, which can promote flowering, *PvPin1* delayed flowering. In addition, *PvPin1* could be regulated by abscisic acid (ABA) and methyl jasmonate (MeJA). *PvPin1* might act as a flowering repressor in bamboo by responding to ABA and MeJA. Our data lay a good foundation for bamboo flowering and provide a basis for understanding bamboo flowering and provide a basis for developing technologies to inhibit it.

## Results

### Isolation of *PvPin1* Gene

**FIGURE 1:**
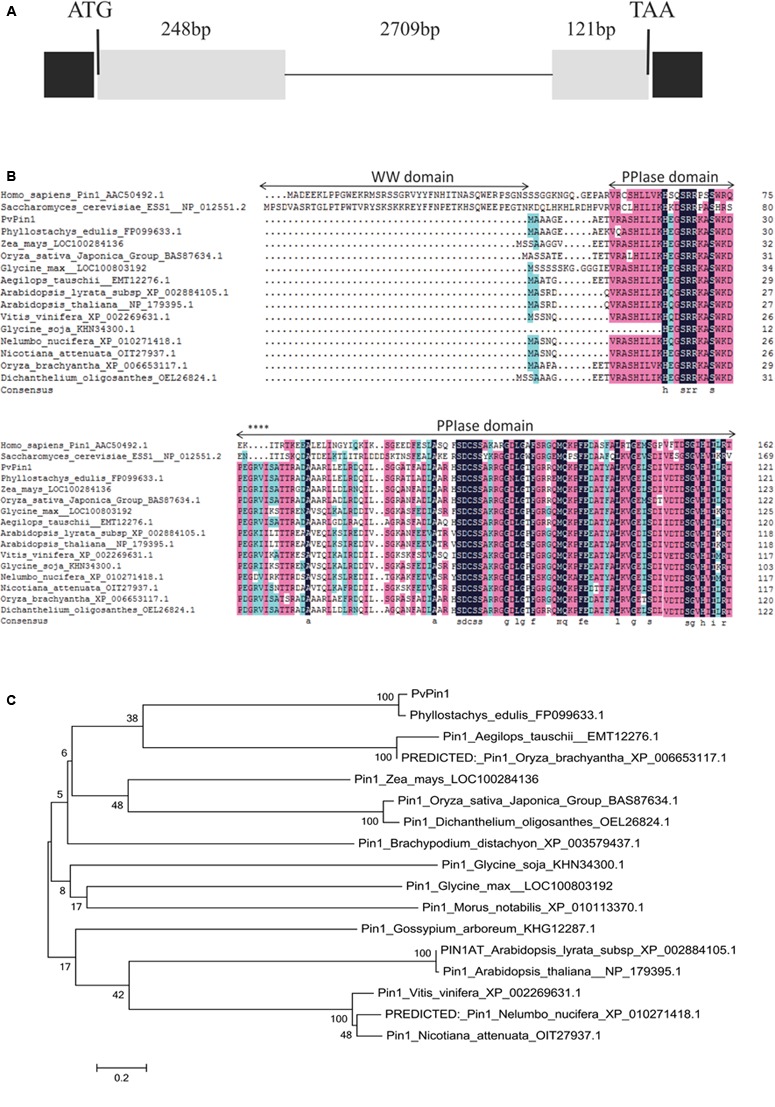
Genomic organization of the *PvPin1*, protein sequence similarities, and phylogenetic analysis of PvPin1 protein. **(A)** Genomic organization of *PvPin1* showing untranslated (black boxes) and translated (gray boxes) regions. **(B)** Alignment of amino acid sequences for PvPin1 and its homologs from other plant species. The PPIase and WW domains are overlined. Asterisks indicate four unique amino acids in plant homologs. **(C)** Phylogenetic analysis of PvPin1 protein. The phylogenetic tree was generated by using MEGA 5.0 and shows branch lengths proportional to distances.

To isolate a *Pin1-like* gene from *P. violascens*, the amino acid sequences of Pin1 homologs from grass family plants were compared and the primers from the conserved regions were designed. Then a 300-bp fragment of the *PvPin1* gene was amplified from *P. violascens.* Using gene-specific primers, a 744-bp cDNA sequence of *Pin1-like* was isolated from *P. violascens* by using 3′ and 5′ rapid amplification of cDNA ends (RACE) and designated as *PvPin1*. DNA sequencing analysis showed that the 744-bp cDNA contained a complete open reading frame (ORF) encoding a polypeptide of 122 amino acids. Based on the cDNA sequence of *PvPin1*, a 3078-bp genomic DNA sequence was cloned. Comparison of the genomic DNA sequence and ORF sequences showed that *PvPin1* had two exons (248 and 121 bp) and one intron (2709 bp) (**Figure 1A**), which was same as *Pin1At* (Landrieu et al., 2000) and *Pin1-like* in rice (NCBI *Oryza sativa* Japonica Group Annotation Release 101). Amino acid sequence alignment revealed that the PvPin1 protein had the theoretical values of 7.97/13148.7 pI/Mw. Secondary structure analysis with SOPMA indicated that the putative PvPin1 protein contained an alpha helix (38.52%), a beta turn structure (16.39%), and a random coil (38.52%). Sequence comparison of the PvPin1 protein with its homologs in other plants showed that the catalytic core was well conserved and contained only a PPIase domain with four additional amino acids (**Figure 1B**).

The sequences of Pin1-like from more than 16 plant species were downloaded from NCBI. Phylogenetic comparison of the PvPin1 protein with homologs in other plants species showed that PvPin1 belongs to the monocots clade and is closely related to Pin1-like proteins from *O. sativa*, *O. brachyantha*, *Dichanthelium oligosanthes*, *Zea mays*, *Aegilops tauschii*, and *Brachypodium distachyon*, especially *Pin1-like* in *P. edulis*, which is the affinis species of *P. violascens*, having the highest identity (94.26%) with *PvPin1* (**Figure 1C**).

### Expression Pattern of *PvPin1*

**FIGURE 2:**
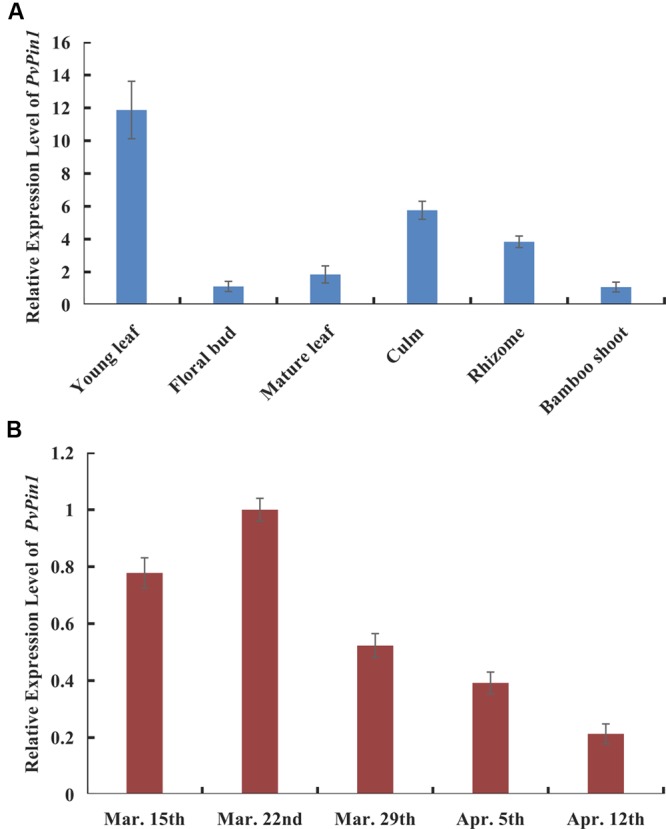
Spatial and temporal expression of *PvPin1* in *P. violascens* by RT-qPCR. **(A)** Relative expression of *PvPin1* in different tissues. **(B)** Relative expression of *PvPin1* in young leaves during flower development. In the first stage (before March 29), the meristem morphology of flower buds was similar with vegetative buds; in the second stage (March 29), the inflorescence had presented, and the floral organs started to take shape; in the third stage (April 5), the floral organs continued to develop and gradually become mature; and in the fourth stage (April 12), the bloom stage, the anther was outcropped from palea (Lin et al., 2012). Data are mean ±SD from three replicates.

RT-qPCR was used to characterize the expression pattern of *PvPin1* in young and mature leaf, floral bud, culm, bamboo shoot, and rhizome tissue in flowering bamboo plants. Although *PvPin1* transcripts were detectable in almost all tested organs, its expression was highest in young leaves and lowest in floral bud (**Figure 2A**).

Also, we used RT-qPCR to detect the temporal expression of *PvPin1* in young bamboo leaves at different flowering stages from March 15 to April 12. *PvPin1* expression peaked on March 22, before flowering. Although the *PvPin1* transcript level increased significantly in young leaves of flowering bamboo plants at the early stage, it declined in leaves during flower development (**Figure 2B**).

### ABRE and TGACG-Motif Elements Exist in Promoters of *PvPin1* and *PePin1* But Not *Pin1At*

**FIGURE 3:**
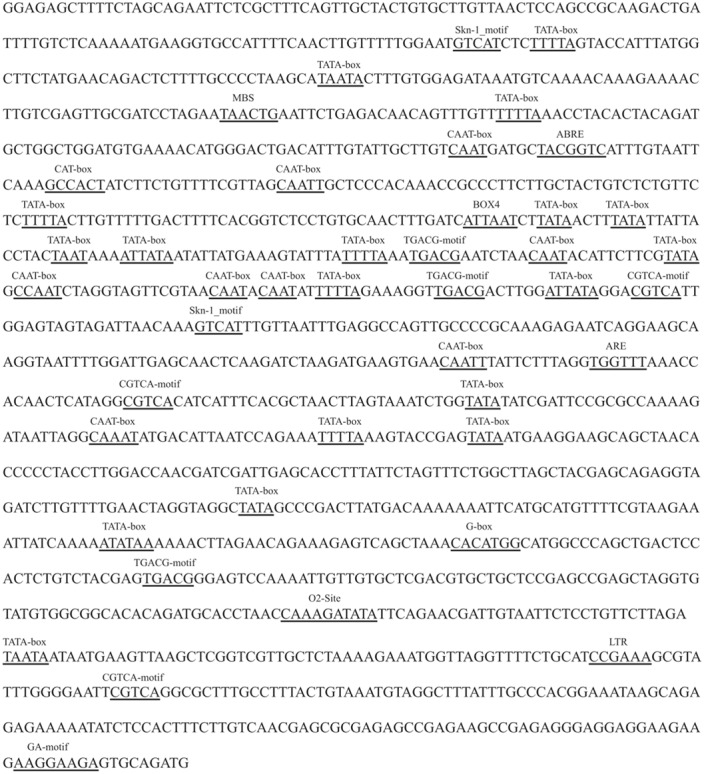
The sequence analysis of the *PvPin1* promoter. Some specific elements are underlined.

To determine whether the expression patterns of *PvPin1*, *Pin1At*, and *PePin1* (*Pin1-like* in *P. edulis*) were associated with the regulation of their promoters, we compared and analyzed their promoter sequences. An upstream 1500-bp sequence of *PvPin1*’s start codon was cloned, and the same length promoter sequences of *Pin1At* and *PePin1* were downloaded from the NCBI database. On sequence alignment, the promoter sequence of *PvPin1* shared 39.09% and 60.43% similarity with those of *Pin1At* and *PePin1*, respectively. The potential *cis*-acting regulatory elements were predicted by using PlantCARE. The typical CAAT-box and TATA-box core elements and other elements involving in light-responsive (LTR), MeJA-responsive (CGTCA-motif), endosperm expression (Skn-1_motif), and anaerobic responsive (ARE) were commonly found in these three promoters (**Figure 3**). However, the ABRE and TGACG-motif *cis*-acting elements, which are regulated by ABA and MeJA, respectively, were specific to the promoter sequences of bamboo (*PvPin1* and *PePin1*).

### ABA and MeJA Treatment Decreased *PvPin1* Expression in Leaf of*P. violascens* Seedlings

**FIGURE 4:**
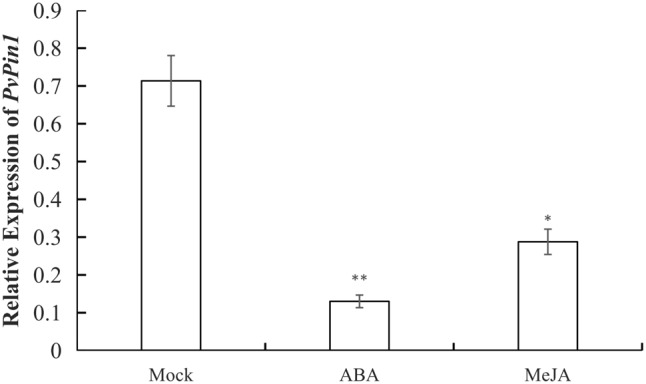
Effect of abscisic acid (ABA) and methyl jasmonate (MeJA) treatments on the expression of *PvPin1* in leaves of *P. violascens* seedlings. Data are mean ± SD. ^∗^*p* < 0.05; ^∗∗^*p* < 0.01 compared to mock, by Student’s *t*-test.

*P. violascens* plants were treated with ABA and MeJA because the ABRE and TGACG-motif elements were specific to the promoter sequences of bamboo (*PvPin1* and *PePin1*). The mRNA level of *PvPin1* in leaf was significantly lower with ABA and MeJA than mock treatment (**Figure 4**), which suggested that *PvPin1* can respond to ABA and MeJA.

### Ectopic Expression of *PvPin1* Delays Flowering Time in *Arabidopsis*

**FIGURE 5:**
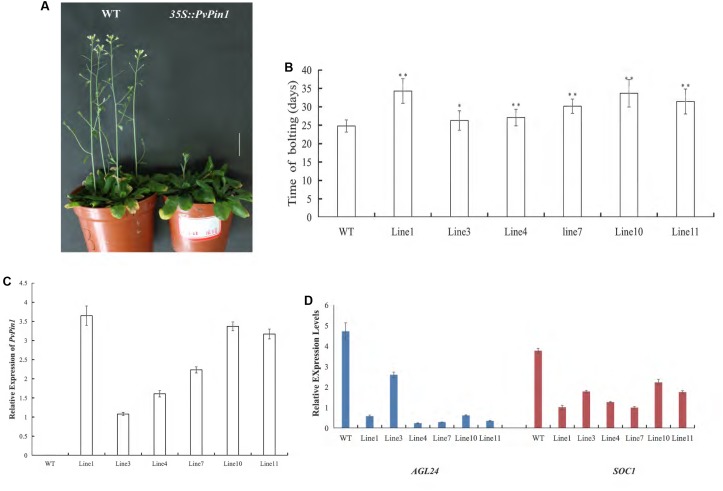
Phenotype analysis of *35S::PvPin1 Arabidopsis* plants under long-day (LD) conditions. **(A)** Late flowering phenotype of transgenic *Arabidopsis*. The scale bar represents 2 cm. **(B)** Days to flowering of T3 transgenic *Arabidopsis* (*n* = 30). **(C)** RT-qPCR expression analysis of *PvPin1*. **(D)** Expression analysis of *AGL24* and *SOC1*. Data are mean ± SD from three replicates. ^∗^*p* < 0.05; ^∗∗^*p* < 0.01 compared to WT, by Student’s *t*-test.

Establishing a regeneration and genetic transformation system in bamboo is difficult (Zang et al., 2016, 2017). To examine the function of *PvPin1* in regulating flowering, we overexpressed *PvPin1* under control of the CaMV *35S* promoter in the *pCAMBIA1301* vector in *Arabidopsis*. Six independent lines in the homozygous T3 generation grown under greenhouse conditions were chosen for further analysis. *35S::PvPin1* transgenic *Arabidopsis* showed a significantly late flowering phenotype (**Figures 5A,B**).

To understand whether the phenotypic alteration of flowering time in transgenic *Arabidopsis* was related to the expression of *PvPin1*, we detected the expression of *PvPin1* in six homozygous lines of *35S::PvPin1 Arabidopsis*. RT-qPCR revealed a positive association between flowering time and the expression of *PvPin1* in transgenic *Arabidopsis* (**Figures 5B,C**).

In *Arabidopsis*, *SOC1* and *AGL24* are important regulatory genes locating at the convergence of the multiple floral induction pathways. We determined the transcript levels of *SOC1* and *AGL24* in transgenic *Arabidopsis* by RT-qPCR. The transcript levels of *SOC1* and *AGL24* in *35S::PvPin1* transgenic plants were greatly decreased (**Figure 5D**). Hence, *PvPin1* delayed the flowering time in *Arabidopsis* by downregulating the expression of *SOC1* and *AGL24*.

### *PvPin1* Overexpression Delays Flowering in Rice

**FIGURE 6:**
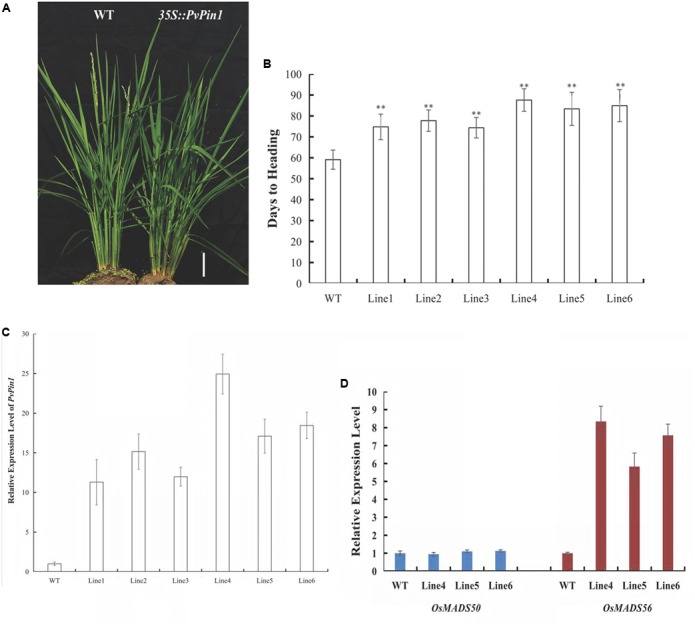
Phenotypic differences between *35S::PvPin1* and WT rice grown in the field. **(A)** Late-flowering phenotype of transgenic rice. The scale bar represents 5 cm. **(B)** Days to heading of T3 homozygous transgenic plants (*n* = 30). **(C)** RT-qPCR expression analysis of *PvPin1*. **(D)** Expression analysis of *OsMADS50* and *OsMADS56*. Data are mean ± SD from three replicates. ^∗∗^*p* < 0.01 compared to WT, by Student’s *t*-test.

To further examine its function, *PvPin1* was transformed into *O. sativa* (Dongjing), a member of the same grass family as bamboo. We analyzed the flowering time in six independent lines in the homozygous T3 generation that were grown under field conditions. *35S::PvPin1* transgenic rice plants showed a significantly late-flowering phenotype (**Figures 6A,B**). Moreover, days to heading were positively associated with the expression of *PvPin1* in transgenic rice (**Figures 6B,C**).

*OsMADS50* and *OsMADS56* are two *SOC1* homolog genes in rice. Because *SOC1* expression was markedly decreased in *35S::PvPin1* transgenic *Arabidopsis* plants, we determined the expression of *OsMADS50* and *OsMADS56* in transgenic rice lines 4, 5, and 6. *OsMADS56* expression was greatly increased in these lines, with no significant difference in expression of *OsMADS50* in comparison with wild-type rice (**Figure 6D**), so overexpression of *PvPin1* inhibited flowering in transgenic rice by upregulating the expression of *OsMADS56*.

### PvPin1 Was Localized in the Nucleus and Cytolemma

**FIGURE 7:**
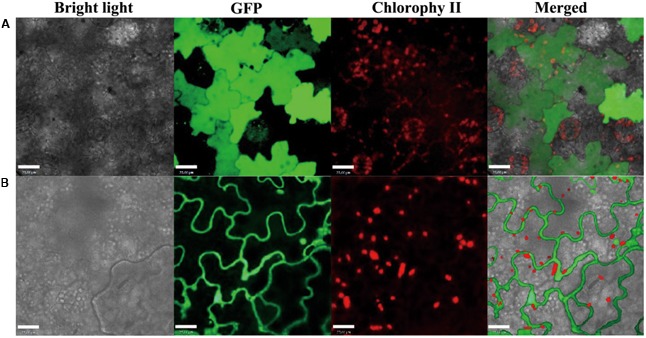
Subcellular localization of PvPin1 in leaf cells of *N. benthamiana*. **(A)** Fluorescence of the control green fluorescent protein (GFP) distributed throughout the cell. **(B)** PvPin1::GFP localized in both the cytolemma and nucleus. Bar = 23 μm.

Pin1At localizes in both the nucleus and cytoplasm (Wang et al., 2010). We used the infection method (Escobar et al., 2003) to determine the subcellular localization of PvPin1 protein. The fusion protein PvPin1-GFP located in the nucleus and cytolemma of epidermal cells of tobacco (*Nicotiana benthamiana*), whereas as a control, the GFP protein distributed in the whole tobacco cells (**Figure 7**). The different localization between *Pin1At* and *PvPin1* implies that they might have different function.

## Discussion

Bamboo usually experiences a long vegetative phase before flowering, so some floral suppressors may be working during this long phase to inhibit bamboo flowering. The inhibiting effect of these flower suppressors could be relieved when bamboo is under stress or undergoing a lengthy vegetative growth. *Flowering locus C* (*FLC*) is an important flower repressor in *Arabidopsis*; however, no *FLC* homologs were determined in monocot plants until now (Doi et al., 2004; Helliwell et al., 2006). Many flowering promoters in bamboo have been reported (Tian et al., 2005; Lin et al., 2009, 2010; Guo et al., 2016; Liu et al., 2016a). We previously showed that *BoTFL1-like* and *PvFRIL* might be possible floral suppressors of bamboo (Zeng et al., 2015; Liu et al., 2016c). In this study, we identified and characterized another possible floral suppressor, a *Pin1-like* gene from *P. violascens* named *PvPin1. PvPin1* was expressed in all tested tissues in bamboo, but its expression was highest in young leaf and lowest in flower bud (**Figure 2A**). *PvPin1* expression peaked before flowering then gradually decreased (**Figure 2B**). Overexpression of *PvPin1* conferred a significantly late flowering phenotype in both greenhouse-grown *Arabidopsis* and field-grown rice. Hence, *PvPin1* might be a flowering repressor in bamboo.

Genome sequence analysis showed that *PvPin1*, *Pin1At*, and *Pin1* homologs in rice and corn have only one intron. Amino acid sequence alignment showed that PvPin1 contains only a C-terminal PPIase catalytic domain with four additional amino acids (**Figure 1B**) like Pin1At (Wang et al., 2010) and other Pin1-like proteins in plant. Therefore, the gene and protein structure of *Pin1-like* in plants are conserved. However, *PvPin1* delaying flowering time in transgenic *Arabidopsis* grown under greenhouse conditions and transgenic rice grown under field conditions, which differs from *Pin1At*, known as a flowering promoter. Furthermore, PvPin1 also has different protein localization and promoter *cis*-elements from *Pin1At*. These differences might be caused by evolutionary diversity of the genes and result in the unique flowering characteristics of bamboo.

**FIGURE 8:**
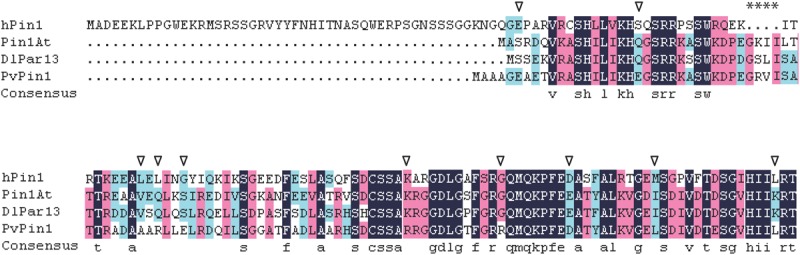
Alignment of amino acid sequences for hPin1 in human, Pin1At, DlPar13 in *Digitalis lanata*, and PvPin1. Asterisks indicate four unique amino acids in plant Pin1 homologs, triangle indicates special amino acid of PvPin1 in comparison with Pin1At and DlPar13.

A protein’s location is mainly determined by its amino acid sequence (Olson et al., 2002). The nuclear localization of hPin1 from human is responsibly directed by the Pin1-WW domain (Rippmann et al., 2000). Recent studies showed that Pin1At from *Arabidopsis* and *DlPar13* from *Digitalis lanata* localized in the nucleus and cytoplasm (Metzner et al., 2001; Wang et al., 2010), and we found that PvPin1 localized in nuclear and cytomembrane. These three plant proteins have no WW domain but have four additional amino acids (**Figure 8**). Whether the four additional amino acids are associated with nuclear localization is unknown. In addition, comparison of protein sequences showed that Pin1At and DlPar13 have the same 10 amino acids, which differs from PvPin1 (**Figure 8**) and may result in their different protein localization and further lead to different functions in flowering regulation.

Multiple genetic pathways coordinately control floral transition in *Arabidopsis* (Koornneef et al., 1998; Levy and Dean, 1998; Mouradov et al., 2002). *SOC1* and *AGL24* are essential regulatory genes involved in multiple floral induction pathways (Lee et al., 2000; Samach et al., 2000; Yu et al., 2002; Michaels et al., 2003; Liu et al., 2007). *OsMADS50* and *OsMADS56* are *SOC1* homologous genes in rice (Nam et al., 2005). *OsMADS50* acts as a flowering activator whose overexpression could promote flowering in transgenic *Arabidopsis* (Tadege et al., 2003). *OsMADS50* and *OsMADS56* may form a complex to delay rice heading time (Ryu et al., 2009). In this study, we found that overexpression of *PvPin1* could downregulate the expression of *AGL24* and *SOC1* in *Arabidopsis* and upregulate *OsMADS56* in rice to delay the flowering time. However, whether the regulation of *SOC1* and *AGL24* in *Arabidopsis* and *OsMADS56* in rice is indirect or direct requires further experiments.

Plant hormones are related to flower development. Lu et al. (2012) showed that flower bud differentiation could be promoted with a high ABA level in *P. violascens*. Abscisic acid could promote flowering by activating the key floral gene *flowering locus T* (*FT*) in *Arabidopsis* (Conti et al., 2014). Methyl jasmonate could also affect flowering time in some other species (Diallo et al., 2014). In this study, ABRE (responding to ABA) and TGACG-motif (responding to MeJA) were found as specific *cis*-acting elements in the promoter of *PvPin1* and *PePin1* (bamboo) in comparison with *Pin1At* (*Arabidopsis*). ABA and MeJA treatments could reduce the expression of *PvPin1* (possible flowering repressor) in *P. violascens*. In addition, ABA and MeJA might promote flowering by upregulating the expression of *PvMADS56* (flower promoter) in *P. violascens* (Liu et al., 2016b). Thus, ABA and MeJA might promote flowering by affecting multiple genes such as *PvMADS56* and *PvPin1* via a complicated regulatory network in bamboo, and their inhibitor may be used for inhibiting bamboo flowering for bamboo forest management.

We found that *PvPin1* is evolutionarily conserved in gene and protein structure in comparison with *Pin1-like* homologs from other plants, especially monocots; however, unlike *Pin1At*, *PvPin1* might be a flowering repressor that can delay bamboo flowering. In addition, our results indicate that ABA and MeJA can significantly reduce the expression of *PvPin1* to promote bamboo flowering. Our results are helpful to disclose the bamboo flowering mechanism and could be used for developing new technologies to inhibit bamboo flowering.

## Materials and Methods

### Plant Materials and Growth Conditions

*P. violascens* samples were collected from the campus of Zhejiang Agriculture and Forestry University. Wild-type (ecotype Columbia) and transgenic plants of *A. thaliana* were cultivated in a controlled temperature room under 22°C with 16-h light/8-h dark. *N. benthamiana* was grown in a controlled temperature room under 28°C with 10-h light/14-h dark. Rice (*O. sativa* cv. Dongjing) plants are cultivated in the field of Lin’an (Zhejiang, China, north latitude 30°14′ and east longitude 119°42′).

### Isolation of *PvPin1* cDNA and Its Intron Sequence from *P. violascens*

Total RNA from *P. violascens* was isolated by using RNAiso Plus (Takara, Shiga, Japan), then Reverse Transcriptase M-MLV (Takara, Japan) was used to synthesize first-strand cDNA. A specific *Pin1-like* cDNA fragment (approximately 300 bp) was amplified by using the pair of primers (Pin1-1 and Pin1-2, **Table 1**), which were designed by comparing the amino acid sequences of Pin1 homologs from grass family plants including *P. edulis* (FP099633.1), *O. rufipogon* (CU406178.1), *O. sativa* (AK243434.1), *Triticum aestivum* (AK333419.1), and *Zea mays* (NM001157033.1). The 3′ end and 5′ partial cDNA of *Pin1-like* were isolated with the RACE kit (Invitrogen) by using gene-specific primers (3′-1 and 3′-2; 5′-1 and 5′-2, **Table 1**). Finally, the full-length ORF sequence was obtained by using the primers (ORF-F and ORF-R, **Table 1**) based on the known 5′ and 3′ sequences.

**Table 1:** Sequences of the primers used in this study.

Primer	Sequences (5′→3′)	Description
Pin1-1	TGCCCACGGAAATAAGCAGAGAG	Primers for conserved sequence
Pin1-2	GAGAGGATCTGGTCGCGGAGTTC	
3′-1	AAAGCCCAACATCGGTATCCAC	Nested gene-specific primers for 3′-RACE
3′-2	GACAATCCAGTGAAGGTGCTCC	
5′-1	ATGTCTAGGTCTGTGGAGCCTC	Nested gene-specific primers for 5′-RACE
5′-2	TCAGCGTCTCCTGGCAGCAGTC	
ORF-F	ATGGCGGCGGCCGGAGAGGC	Primer pairs for ORF
ORF-R	TTAGGCAGTCCGCAGGATGATGTGA	
S1-F	TCCGACTACATTGAGGGGTT	Nested gene-specific primers for promoter sequence
S1-R	GAAGGTGGCTGCCGGAGAGGATCTG	
S2-F	GGAGAGCTTTTCTAGCAGAA	
S2-R	GAGATGACGCGGCCCTCGGGGTCCTTC	
PeUBC18-F	CGGGCCTCGCACATCCTTAT	Primer pairs used for quantitative real-time PCR
PeUBC18-R	CGCCAACCTTGAGTGCATATGTG	
qPCR-F	CGGGCCTCGCACATCCTTAT	
qPCR-R	CGCCAACCTTGAGTGCATATGTG	
AGL24-F	GAGGCTTTGGAGACAGAGTCGGTGA	
AGL24-R	AGATGGAAGCCCAAGCTTCAGGGAA	
SOC1-F	AGCTGCAGAAAACGAGAAGCTCTCTG	
SOC1-R	GGGCTACTCTCTTCATCACCTCTTCC	
TUB2-F	ATCCGTGAAGAGTACCCAGAT	
TUB2-R	AAGAACCATGCACTCATCAGC	
OsMADS50-F	AAAGCTGACGCTGATGGTTTG	
OsMADS50-R	GTTTCGACATCCATGTTGTC	
OsMADS56-F	GACCGCTATAAAGCATACACA	
OsMADS56-R	TCATGTGGTTAGCCACCAGC	
Ubiquitin-F	CACGGTTCAACAACATCCAG	
Ubiquitin-R	TGAAGACCCTGACTGGGAAG	


Genomic DNA was isolated by the modified CTAB method (Reichardt and Rogers, 1993) from leaves. Then a 2709-bp intron sequence of *PvPin1* was obtained by using the primers ORF-F and ORF-R.

### Isolation of *PvPin1* Promoter from *P. violascens*

The sequence of *PvPin1* ORF was used for a BLAST search in the transcript online database for *P. edulis* (affinis species of *P. violascens*) (Peng et al., 2013)^1^. A sequence (ID: FP099633.1) that exists between PH01001300G0520 and PH01001300G0540 with the highest identity to *PvPin1* was identified. Then the correlative genomic sequence in PH01001300 was extracted from the genome database of *P. edulis* (Peng et al., 2013) and used to design the primers (S1-F, S1-R; S2-F, S2-R, **Table 1**) for amplifying the promoter of *PvPin1*. A promoter sequence of 1500 bp was obtained from the DNA by using Nested PCR (Gundersen and Lee, 1996).

### Expression Pattern of *PvPin1*

The RT-qPCR primers (qPCR-F and qPCR-R, **Table 1**) were designed by using the full-length ORF sequence of *PvPin1*. Here, *PeUBC18* was used as the internal control gene (Qi et al., 2013; Liu et al., 2016b; **Table 1**) because of the close relationship between *P. edulis* and *P. violascens*. CFX96TM Real-Time PCR Detection System (Bio-Rad) and the SYBR Premix ExTaq II mix (Takara) were used for PCR amplification. The program was 95°C for 3 min, followed by 40 cycles of amplification (95°C for 15 s, 60°C for 30 s). Reactions were performed in 20-μl mixtures consisting of 10 μl 2× SYBR Premix Ex Taq II Mix, 0.5 μl each of forward or reverse primer, 1 μl cDNA template (50 ng/μl), and 8 μl double distilled H_2_O (Liu et al., 2016b). The data were analyzed by the 2^-ΔΔC_*t*_^ method (Livak and Schmittgen, 2001).

### Binary Plasmid Construction and Analysis of Transgenic Plants

The full-length ORF for *PvPin1* was cloned into the binary vector *pCAMBIA1301* under the control of the Cauliflower mosaic virus (CaMV) *35S* promoter. Recombinant vector was transferred into *A. tumefaciens* strain GV3101, then into *Arabidopsis* by the floral dip method (Clough and Bent, 1998). Transformants were screened in media with 50 μg/ml kanamycin. The same construct was also transformed into rice plants (Dongjing) mediated by *A. tumefaciens* strain EHA105 as described (Xu et al., 2017). Positive transgenic rice lines were confirmed by genomic PCR. The expression of *SOC1* and *AGL24* genes in transgenic *Arabidopsis* in six T3 lines and WT *Arabidopsis*, and the expression of *OsMADS50* and *OsMADS56* in transgenic rice in three T3 lines and WT rice were analyzed by real-time qPCR with gene-specific primers (**Table 1**) following the protocol in expression pattern of *PvPin1* section “Expression Pattern of *PvPin1*.”

### Subcellular Localization of PvPin1

The full-length coding sequence without terminator codon (TAA) of *PvPin1* was cloned into the CaMV *35S-GFP* vector that allowed the system to generate a PvPin1-GFP fusion protein for investigating subcellular location in epidermal cells from tobacco (*N. benthamiana*) and the transient expression assay method (Escobar et al., 2003) was adopted. The tobacco epidermal cells were visualized on confocal laser scanning microscopy (LSM510, Zeiss, Germany).

### Bioinformatics Analysis

A BLAST search in the NCBI database was used to obtain the protein sequences of Pin1-like. The phylogenetic tree was constructed by the neighbor-joining method with the parameter bootstrap (10,000 replicates) in MEGA 5.0. The software ProtParam from ExPASy^2^ was used to obtain the molecular weights (MW) and theoretical isoelectric point (pI) of PvPin1 protein. PlantCARE (Lescot et al., 2002) was used to analyze *cis*-acting regulatory elements in the *PvPin1* promoter.

### ABA and MeJA Treatment

Leaves of *P. violascens* seedlings were sprayed with ABA (100 μM), MeJA (100 μM), and water as a blank control once a day for 9 days. Every treatment was performed with three biological replicates. The *PvPin1* transcript level was detected after treatment.

## Author Contributions

XL, ZZ, and XY conceived and designed the experiments; YF contributed materials of transgenic rice; XL and LZ monitored the experimental work; ZZ, XY, and HW performed the experiments; ZZ analyzed the data; and ZZ and XL wrote the paper.

## Conflict of Interest Statement

The authors declare that the research was conducted in the absence of any commercial or financial relationships that could be construed as a potential conflict of interest.

## Footnotes

1 http://www.bamboogdb.com

2 http://exPasy.org
